# A Remarkable Case of Desquamation

**Published:** 1890-12

**Authors:** R. W. Skipper

**Affiliations:** Holley, Texas


For Daniel's Texas Medical Journal.

### A REMARKABLE CASE OF DESQUAMATION.

BY R. W. SKIPPER, M. D., HOLLEY, TEXAS.

ON JANUARY last I was asked to treat a girl, Carr, colored, and her father gave the following history of the case. I did not see her on account of pressing engagements. Patient was thirteen years of age and very strong. She was sick with a fever of remittent type and had severe pains in the abdomen. Her father said that she had the "hottest skin" that he ever saw, and it was constantly dry for four days, spite of best methods to reduce fever. She had a "course" of mild mercury daily, and quinine abundantly at night. On the third day she passed a lumbricoid worm and pains in the abdomen grew worse. I gave santonine at once with hydr. chlor. mite, and in two days she had passed thirty-six of the largest worms.

By this time the skin was moist, and convalescence began and was soon complete. Extensive desquamation took place, the cuticle coming off in large patches. She is now, November 7, quite well, and has been well from that time.

Could the worms in this case have caused the desquamation of cuticle which was complete? She had taken quinine repeatedly, without showing any such idiosyncracy. It is for this reason that I report this case. The "gloves and slippers," as I call the perfect casts of the hands and feet which were shed, are in possession of Drs. Daniel and Brooks, the Journal men of Texas, and may be seen by any that will call upon them.

